# The effect of leg-to-body ratio on male attractiveness depends on the ecological validity of the figures

**DOI:** 10.1098/rsos.170399

**Published:** 2017-10-18

**Authors:** Thomas M. M. Versluys, William J. Skylark

**Affiliations:** Department of Psychology, University of Cambridge, Cambridge CB2 3EB, UK

**Keywords:** human mate choice, male attractiveness, limb proportions, leg-to-body ratio

## Abstract

Leg-to-body ratio (LBR) predicts evolutionary fitness, and is therefore expected to influence bodily attractiveness. Previous investigations of LBR attractiveness have used a wide variety of stimuli, including line drawings, silhouettes, and computer-generated images based on anthropometric data. In two studies, community samples of heterosexual women from the USA rated the attractiveness of male figures presented as silhouettes and as detailed computer-generated images with three different skin tones (white, black, and an artificial grey). The effects of LBR depended on the image format. In particular, a curve-fitting analysis indicated that the optimally-attractive LBR for silhouettes was fractionally below the baseline, whereas the optima for more detailed computer-generated images was approximately 0.5 s.d. above the baseline and was similar for all three skin-tones. In addition, the participants' sensitivity to changes in the LBR was lowest for the silhouettes and highest for the grey figures. Our results add to evidence that the most attractive LBR is close to, but slightly above, the population mean, and caution that the effects of limb proportions on attractiveness depend on the ecological validity of the figures.

## 1. Introduction

The role of attractiveness in shaping life outcomes and human well-being is well established. More attractive people tend to receive favourable treatment in employment [1,2], electoral [3,4], and judicial decisions [5], earn higher salaries [6,7], have longer and more stable marriages and sire more children [6,8–10]. They are also judged to be more sociable [11], intelligent [12] and healthy [13], a set of attributions known as ‘the halo effect’ [14]—although there are contexts in which physical attractiveness may be disadvantageous [15,16].

There is long-standing interest in the relationship between body morphology and attractiveness. From a biological perspective, traits are attractive when they signal ‘biological fitness’ (or just ‘fitness’), defined as an organism's ability to survive and reproduce in its environment [17,18]. Research has identified many traits in the face and body that contribute to attractiveness [19–21], but relatively little is known about the role of limb proportions, despite good theoretical reasons for believing that limbs signal fitness. Like the face and body, they can reflect underlying developmental and genetic conditions in an organism, displaying deviations from average or varying degrees of bilateral symmetry. They can also be subject to surplus growth, contributing to the body's reserve tissue [22]. A direct link to fitness is also apparent, with relatively short legs (defined by the ratio to total height) being correlated with negative health outcomes such as poor insulin resistance and coronary heart disease [23,24], high blood pressure, cholesterol and body mass index [25], diabetes mellitus [24,26], and dementia [27,28]. Conversely, excessively long legs are associated with harmful genetic diseases, such as Marfan syndrome [29,30]. Slightly above-average legs, on the other hand, have been linked to developmental stability, good nutrition, and high socioeconomic status [22].

Despite these links between limb proportions and fitness, existing research on the relationship between limb proportions and attractiveness is limited and has produced inconclusive findings. Our focus is on male leg-to-body ratio (LBR), typically defined as leg length divided by total height (e.g. [31]). Early studies indicated that men with low LBRs are more attractive [32,33], but more recent studies have found that average [34,35] or above-average [31,36] proportions are preferable. Other research has found that optimum male LBR varies cross-culturally [37], with no detectable preference at all in some cases [38]. A similar diversity has been found in studies of female figures [31–35,37,39–41].

These mixed results might be owing to the varied and sometimes sub-optimal stimuli used in the experiments. Some studies have selected LBRs without reference to biological data [32,33,42]; others selected a baseline corresponding to the mean of a relevant population [31,34–38], albeit with different approaches to choosing LBRs on either side of the baseline (e.g. [31,36,40]). In addition to the consequences for ecological validity of some of these choices, the use of different LBRs in different studies is potentially important because most researchers have focused on identifying which of the presented stimuli was most attractive (e.g. by running ANOVA with follow-up tests), such that the optimal LBR is constrained to be one of the (study-specific) tested values.

More importantly, previous studies of LBR preferences have used widely varying image formats, including line drawings [32,33], stick figures [42], handmade figurines [36], and black silhouettes adapted from photographs [31,35,37,38]. Some of these necessitated relatively crude image-manipulation techniques that introduced anatomical distortions such as compression of the hands and feet—aspects of morphology known to influence attractiveness [43–45]. More recent research has used realistic computer generated images (CGI) of people with normal skin pigmentation [34] or in black-and-white [40].

This cross-study variation is important because different figure types can induce different preferences. In particular, Kościński [20] found that the most attractive waist-to-hip ratio differed for realistic colour images and black silhouettes of the same proportions. Correspondingly, it has been argued that researchers should avoid silhouettes and other artificial stimuli because they lack ecological validity (e.g. [46]). However, whether the presentation format actually modulates the link between limb ratio and attractiveness is an open question.

The present research has two aims. First, it examines the effect of LBR on male attractiveness using a methodology that rectifies many of the problems discussed above: the stimuli were based on a validated anthropometric database with LBRs that were 0, 1, 2 and 3 standard deviations above/below the mean, reflecting realistic variation in the population; the stimuli were created using advanced design software to maximize realism; and a curve-fitting approach [34] was used to estimate both the optimum LBR and the sensitivity to departures from that optimum. Second, it tests the generality of LBR preferences across changes in image format. Rather than focusing on the formats used in early studies, we examine four options available to researchers using contemporary CGI software: black silhouettes [35] and three types of three-dimensional-rendered figure, one with white (‘Caucasian’) pigmentation [34], one with black (e.g. African-American) pigmentation, and one with non-naturalistic greyscale (black and white) colouring [40].

## 2. Method

We conducted two studies; study 2 was a direct replication of study 1 and so the studies are described together.

### 2.1. Stimuli

The stimuli were realistic CGI human male figures created using state-of-the-art design software (Daz Studio 4.9: https://www.daz3d.com/daz_studio) with the Male Anatomy add-on package. The software provides a default, anatomically-accurate model (‘Michael 3’) which can be modified by specifying precise values for the skeletal dimensions (bone lengths) to give fine control over the limb proportions.

Our selection of limb proportions was based on a database of male participants in the 1988 US Army Anthropometry Survey (ANSUR) [47], which provides 132 standard anthropometric measurements from approximately 9000 United States (US) military personnel. There are no absolute rules in traditional anthropometry regarding how leg length is defined (for reviews, see [22,48]), so the most appropriate measurements were selected from the database. Specifically, total leg length was measured as the height to the trochanter landmark on the hip minus ankle height, which was calculated as the distance from the floor to the lateral malleolus landmark on the ankle. This is a closer approximation of anatomical leg length than the more common measurement of height to the perineum (crotch) seen in the literature [22]. The LBR was calculated by dividing leg length by total height (measured from the base of the heel to the top of the head). The mean LBR was 0.491 with a standard deviation of 0.015.

**Figure 1.**
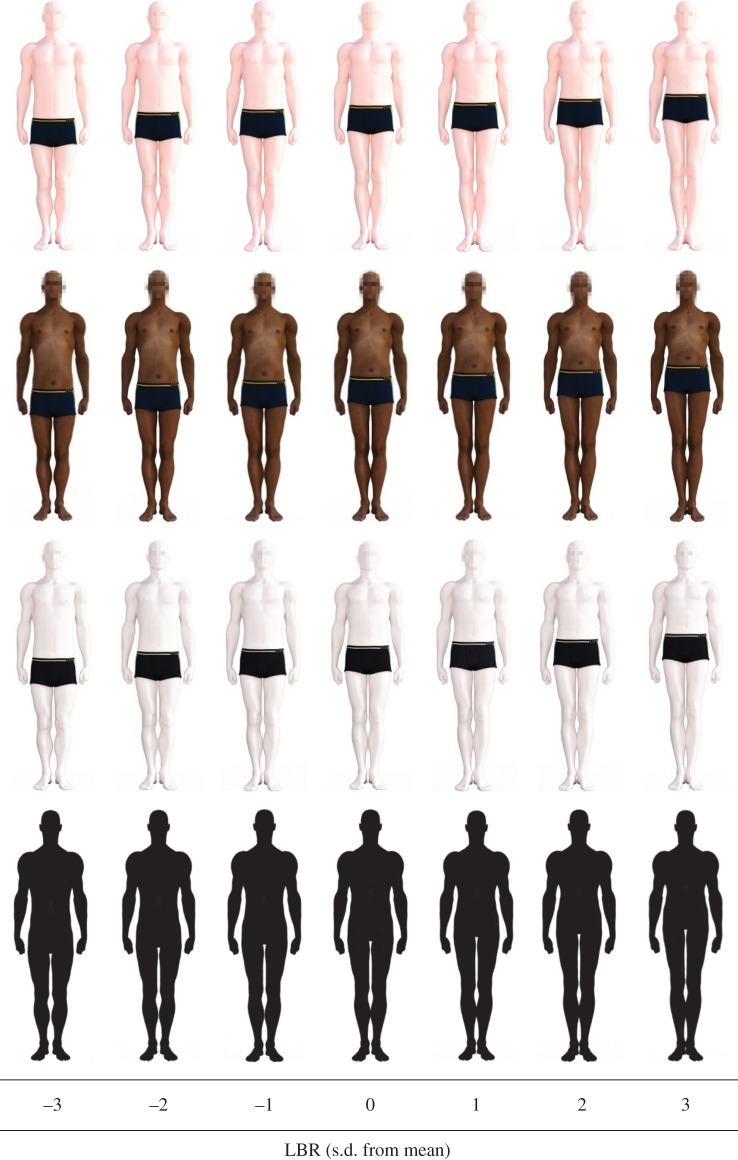
The figure stimuli. From top to bottom, the rows show the white, black, grey and silhouette formats.

A baseline figure was created using the proportions of the original model provided in Daz Studio (‘Michael 3’), with nominal height set at the database male average of 175 cm and LBR set at the database average of 0.491. The other stimuli were constructed to have the same height, with LBRs that were ±1, 2 or 3 s.d. from the mean, such that the tested LBRs were: 0.447, 0.462, 0.477, 0.491, 0.506, 0.521 and 0.535. Maintaining a fixed height required changes to torso size as leg length varied, with proportional alterations in the thorax and abdomen. The faces of stimuli were pixelated to prevent facial features from dominating attractiveness judgements or interacting with limb proportions. The figures are shown in figure 1; the absolute dimensions and visual angles of the stimuli were variable because they were presented on participants' own computers.

### 2.2. Design and procedure

The studies were conducted online and had a 4 (image format) × 7 (leg-length) within-subject design. The study was advertised as being for female participants and the first page of the task asked the participant's gender; those who indicated male were redirected away from the survey. After providing informed consent, participants were told that they would be asked to judge the attractiveness of male figures. They were told that some of the figures were similar to one another but that they were all slightly different, that there were no right or wrong answers, and that they should answer honestly. Each figure was presented on a separate webpage and participants rated its attractiveness on a scale from 1 (‘not at all’) to 7 (‘very much so’). They judged a total of 28 figures, one for each combination of image-format and leg-length, in random order. Finally, they reported demographic information: ethnic origin (White American; White other; Black/African American; Black other; Hispanic; Asian; Native American; Pacific Islander; other); sexuality (straight or heterosexual; gay or lesbian; bisexual; other; prefer not to say); and age (indicating with a slider ranging from 0 to 100).

The task, stimuli and procedure were identical in studies 1 and 2.

### 2.3. Participants

For study 1, an *a priori* power analysis indicated that a sample size of 40 would give more than 95% power to detect a ‘medium’ effect (*f* = 0.25) for a main effect of image format; we intended to ‘overshoot’ that minimum. We recruited heterosexual women from the United States via Amazon's Mechanical Turk, an online platform which more closely approximates the US population than many convenience samples [49] and which produces data of comparable quality to laboratory studies [50]. In keeping with previous work, we only took the first occurrence of a given ip address and only included participants who completed the task and did not report problems viewing the images in the study [51]. Only participants who answered ‘straight or heterosexual’ to the sexuality question were included in the final sample, which comprised 74 women aged 20–69 (*M* = 36.4, s.d. = 10.4), ethnicities: White American (67.6%); White other (2.7%), Black/African American (14.9%), Asian (10.8%), Hispanic (2.7%), other (2.7%).

For study 2 we recruited a fresh sample of participants from the same population as study 1. A power calculation indicated that a sample of 100 would give more than 99% power to detect a format × LBR interaction of the same size as that found in study 1. Our final sample comprised 112 women aged 22–67 (*M* = 37.8, s.d. = 11.6), ethnicities: White American (84.8%), White other (0.9%), Black/African American (8.0%), Asian (4.5%), Hispanic (0.9%), other (0.9%).

## 3. Results

**Figure 2.**
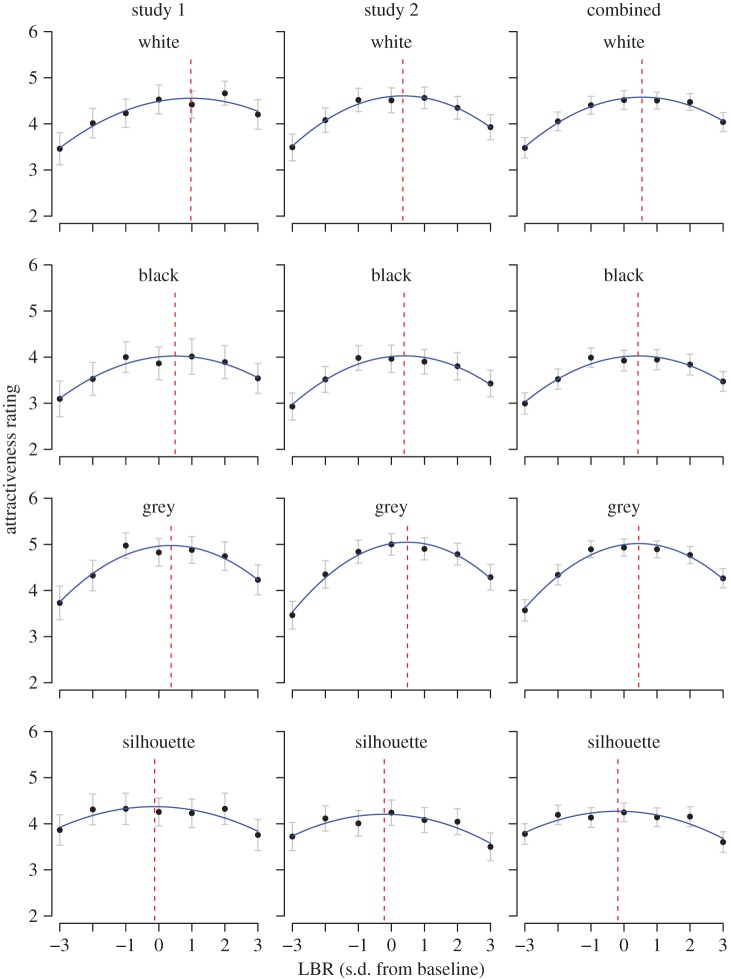
Mean attractiveness ratings for each LBR for each stimulus type; the left column shows the results from study 1; the central column shows the results from study 2; the right column shows the results pooled across the two studies. Error bars are 95% confidence intervals. The blue lines are the best-fitting quadratic curves through the mean judgements for each panel. The dashed red lines show the optimally-attractive LBR values, estimated from the regression coefficients.

The mean attractiveness ratings for each combination of LBR and image format are plotted in figure 2, which shows the data for studies 1 and 2, and after combining the data from the two studies.

For study 1, a 7 × 4 within-subject ANOVA indicated that attractiveness depended on both LBR, *F*_4.12, 300.56_ = 26.62, *p* < 0.001, $\eta_{p}^{2}=0.267$, and image format, *F*_2.30,167.97_ = 15.03, *p* < 0.001, $\eta_{p}^{2}=0.171$, and the effects of LBR were modulated by the image format, *F*_15.81, 1153.96_ = 3.48, *p* < 0.001, $\eta_{p}^{2}=0.05$ (here and throughout, a Huynh–Feldt correction was because of violations of sphericity). Bonferroni-corrected follow-up tests established that the grey figures had the highest mean attractiveness ratings (*M* = 4.53, s.d. = 1.01) and were more attractive than all other formats (all corrected ps≤0.021); the black figures received lower ratings than all other formats (*M* = 3.70, s.d. = 1.56, all ps≤0.003); the white figure and silhouette were similarly attractive (*M* = 4.15, s.d. = 1.13 and *M* = 4.22, s.d. = 1.05, respectively, *p* = 1.000).

Study 2 produced the same effects: a main effect of image format, *F*_2.45, 272.00_ = 29.97, *p* < 0.001, $\eta_{p}^{2}=0.21$, a main effect of LBR, *F*_3.78, 419.27_ = 38.81, *p* < 0.001, $\eta_{p}^{2}=0.26$, and an interaction between the two, *F*_16.38, 1817.79_ = 4.76, *p* < 0.001, $\eta_{p}^{2}=0.04$. The grey figures had the highest mean attractiveness ratings (*M* = 4.52, s.d. = 1.03) and were judged more attractive than all other formats (all corrected *p*s < 0.001); the black figures received lower ratings than all other formats (*M* = 33.65, s.d. = 1.24, all ps≤0.015); the white figures and silhouettes received similar ratings (*M* = 4.21, s.d. = 1.05 and *M* = 3.96, s.d. = 1.23, respectively, *p* = 0.084).

To test the consistency of the results across the two studies, we combined the data from studies 1 and 2 and re-ran the ANOVA including study and its interactions with the other factors. There was no overall effect of study, *F*_1,184_ = 0.23, *p* = 0.632, $\eta_{p}^{2}=0.00$, and study did not modulate any of the other effects (study × format: *F*_3,552_ = 0.62, *p* = 0.602, $\eta_{p}^{2}=0.00$; study × LBR: *F*_6, 1104_ = 0.81, *p* = 0.565, $\eta_{p}^{2}=0.00$; study × format × LBR: *F*_18, 3312_ = 1.22, *p* = 0.236, $\eta_{p}^{2}=0.01$). As one would expect from the individual study results, there was a main effect of format, *F*_2.39, 440.42_ = 41.49, *p* < 0.001, $\eta_{p}^{2}=0.18$, a main effect of LBR, *F*_3.96, 728.55_ = 60.15, *p* < 0.001, $\eta_{p}^{2}=0.25$; and a format × LBR interaction, *F*_17.02, 3131.10_ = 6.62, *p* < 0.001, $\eta_{p}^{2}=0.03$.

To gain deeper insight into the effects of limb length and image format, we fit the following regression equation:

$$\text{attractiveness}=\beta_{0}+\beta_{1}\text{LBR}+\beta_{2}\text{LB}{\text{R}}^{2}+e,$$

where attractiveness is the mean attractiveness rating for a given LBR and *e* is the residual error. The blue lines in figure 2 show the best-fitting curves, which were fitted separately for each image format and which describe the data well. The regression coefficients are shown in table 1, and were used to estimate: (i) the optimum LBR for each format (i.e. the location of the peak of the curve), given by $\text{optimum}=-\beta_{1}\text{/}2\beta_{2}$, and (ii) the sensitivity of the participant to deviations from this optimum, given by the absolute value of the second derivative, $\left|2\beta_{2}\right|$(larger values indicate that the judged attractiveness declines more rapidly to either side of the maximal value). We also computed bootstrapped confidence intervals to gauge the uncertainty in these estimates [52,53]. Specifically, for study 1 we sampled with replacement from the original set of participants (the random sample was the same size as the original sample), analysed the resulting data just as we analysed the original data (including computing the differences between the optima/sensitivities for each pair of conditions), and repeated this process 2000 times to obtain percentile-based 95% confidence intervals (the bias-corrected accelerated intervals were virtually identical to the percentile-based values). We took the same approach for study 2 and for the combined data.

**Table 1.** Regression coefficients for each format in each study. Note: The lines were fit to the mean judgements for each LBR in each condition and are plotted in figure 2. The values in brackets are the 95% confidence intervals for the regression coefficient.

		study 1		study 2		combined	
format	term	*B*	*p*	*B*	*p*	*B*	*p*
white	intercept	4.49 [4.29, 4.69]	<0.001	4.59 [4.49, 4.70]	<0.001	4.55 [4.46, 4.64]	<0.001
	LBR	0.13 [0.07, 0.20]	0.005	0.07 [0.03, 0.10]	.005	0.09 [0.06, 0.12]	<0.001
	LBR^2^	−0.07 [−0.11, −0.03]	0.008	−0.10 [−0.12, −0.08]	<0.001	−0.09 [−0.10, −0.07]	<0.001
		*R*^2^-adj = 0.898		*R*^2^-adj = 0.975		*R*^2^-adj = 0.978	
black	intercept	4.01 [3.84, 4.17]	<0.001	4.01 [3.87, 4.16]	<0.001	4.01 [3.87, 4.15]	<0.001
	LBR	0.07 [0.02, 0.13]	0.019	0.07 [0.02, 0.12]	0.013	0.07 [0.03, 0.12]	0.012
	LBR^2^	−0.08 [−0.11, −0.04]	0.003	−0.09 [−0.12, −0.07]	<0.001	−0.09 [−0.11, −0.06]	<0.001
		*R*^2^-adj = 0.904		*R*^2^-adj = 0.947		*R*^2^-adj = 0.942	
grey	intercept	4.96 [4.75,5.17]	<0.001	5.02 [4.88, 5.15]	<0.001	4.99 [4.85, 5.14]	<0.001
	LBR	0.08 [0.01,0.15]	0.031	0.12 [0.08, 0.17]	0.002	0.11 [0.06, 0.15]	0.003
	LBR^2^	−0.11 [−0.15, −0.07]	0.002	−0.12 [−0.15, −0.10]	<0.001	−0.12 [−0.14, −0.09]	<0.001
		*R*^2^-adj = 0.918		*R*^2^-adj = 0.976		*R*^2^-adj = 0.969	
silhouette	intercept	4.37 [4.14, 4.60]	<0.001	4.20 [4.00, 4.41]	<0.001	4.27 [4.07, 4.46]	<0.001
	LBR	−0.01 [−0.09, 0.06]	0.638	−0.03 [−0.09, 0.04]	0.330	−0.02 [−0.09, 0.04]	0.402
	LBR^2^	−0.05 [−0.10, −0.01]	0.027	−0.06 [−0.10, −0.02]	0.012	−0.06 [−0.10, −0.02]	0.012
		*R*^2^-adj = 0.625		*R*^2^-adj = 0.758		*R*^2^-adj = 0.752	

**Figure 3.**
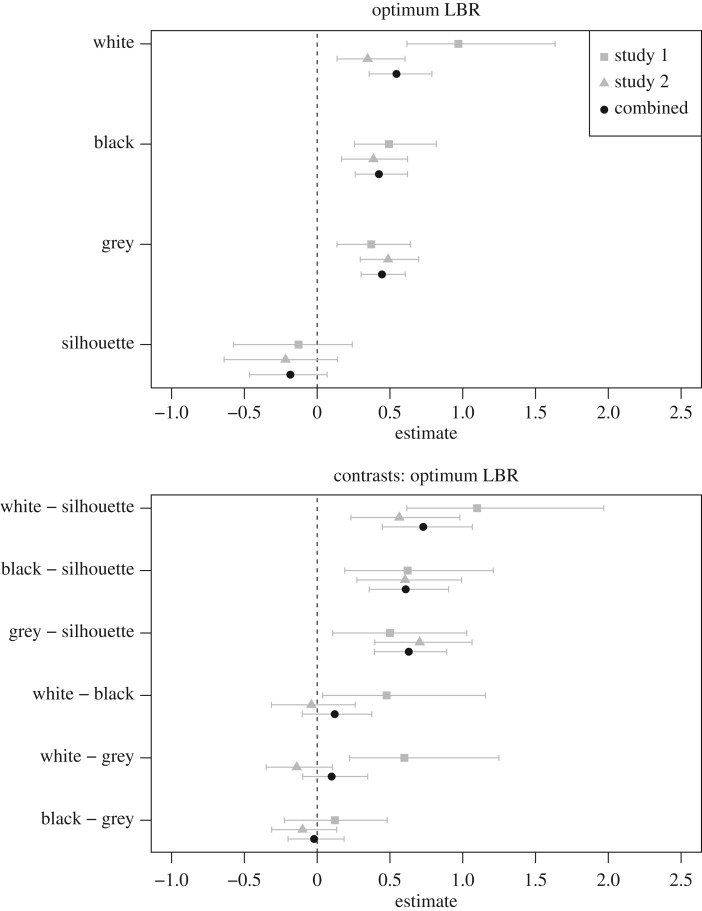
The top panel shows the optimally-attractive LBRs for each format for studies 1 and 2, and with the data from the two studies combined. The bottom panel shows the contrasts between each pair of conditions; for example, the top row shows the optimum for the white figures minus the optimum for the silhouette figures. Error bars are 95% boot-strapped confidence intervals.

The optima are shown as red lines in figure 2 and are plotted in the top panel of figure 3; the combined data are ‘highlighted’ with black symbols because they provide the best estimate of the population values. The most attractive LBRs for the white, black and grey figures are all approximately 0.5 s.d. above the baseline (for the pooled data the values are 0.54, 0.42 and 0.45, respectively); the optimum LBR for the silhouettes is fractionally below the baseline (−0.18 for the pooled data). The bottom panel of figure 3 shows the comparisons between conditions: the white, black and grey formats all have optima that are larger than the silhouettes and are similar to one another, although there is some indication that, in study 1, the optimum is slightly larger for the white figures than for the other two formats.

**Figure 4.**
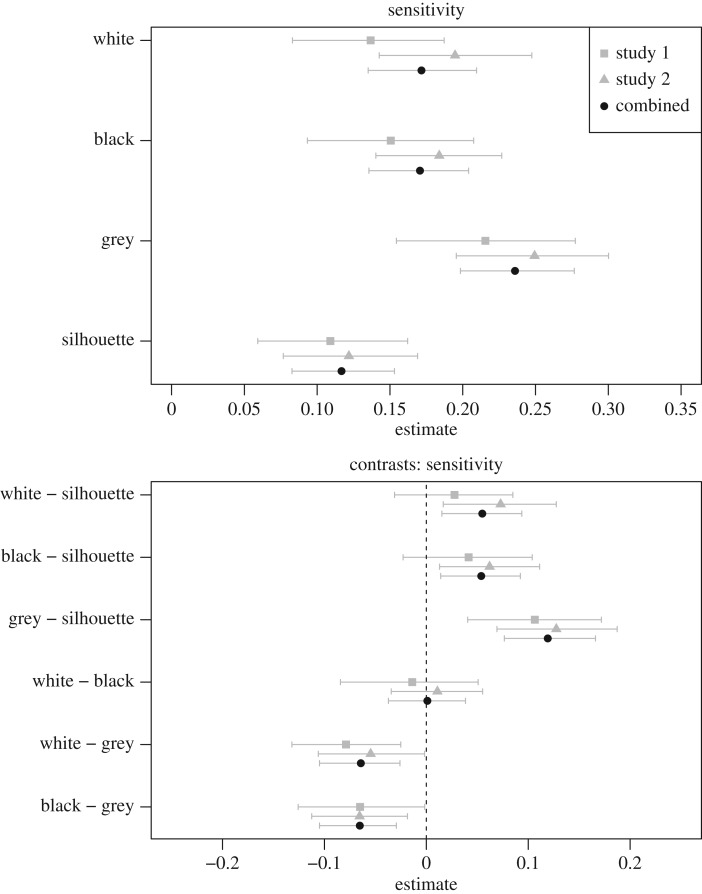
The top panel shows the sensitivity estimates for each format for studies 1 and 2, and with the data from the two studies combined. The bottom panel shows the contrasts between each pair of conditions. Error bars are 95% boot-strapped confidence intervals.

The sensitivity estimates (which, by definition, are all positive) are plotted in the top panel of figure 4; the bottom panel shows the contrasts between each pair of conditions. Sensitivity was higher for the grey figures than for other formats, and was lowest for the silhouettes; the black and white formats were very similar. This pattern is evidenced in the curves shown in figure 1, which are notably flatter for silhouettes and steeper for grey figures.

In summary, the choice of image format affected: (i) the overall attractiveness of the figures, (ii) the leg-to-body ratio that is judged most attractive, and (iii) participants' sensitivity to deviations from that optimum.

## 4. Discussion

Our studies produced two key findings. First, that the effects of leg-to-body ratio on male attractiveness depend on the format of the figures; in particular, both the optimum LBR and the sensitivity to deviations from that optimum are lower for silhouettes than for more realistic rendered figures. Second, that among the realistic images the optimum LBR is about 0.5 s.d. above the population mean and was similar for black, white, and grey figures—although the judgement function was somewhat steeper for the latter. The value of approximately 0.5 s.d. corresponds to the 69th percentile of the population, but is only about 1.5% above the baseline.

Our finding that attractiveness judgements vary with image format echoes recent evidence that the optimally-attractive female waist-to-hip ratio is different for silhouettes and for realistic colour images [20], and supports the broader argument that researchers should focus on naturalistic images when investigating the links between morphology and sexual selection [34,40,46]. One explanation for why the optimum LBR was different for silhouettes is that this format obscures morphology, meaning that people cannot readily judge leg-length relative to other anatomical landmarks; for example, the division between torso and leg may be harder to detect. This would be consistent with our finding that people were also less sensitive to changes in LBR for the silhouette figures, but remains a speculative suggestion. Similarly, we can only speculate as to why there was an overall effect of figure format on attractiveness judgements: the relatively low attractiveness of black figures might be attributed to an own-race preference among our predominantly white participants, but this would not explain why grey figures were judged most attractive overall in both studies. In any case, the main implication of our findings regarding image format, when coupled with those of other researchers, is that previous studies of LBR that used drawings or silhouette stimuli should be treated with caution.

Few previous studies of LBR preference have used anatomically-detailed figures. The study most similar to our own is that by Kiire [34], who used three-dimensional-rendered images and found an optimum very close to the baseline value of 0.457 (the average for Japanese people in their 20s, and somewhat lower than the baseline used in the current studies). This is slightly different from our finding that, for heterosexual US women, the optimally-attractive LBR is about half a standard deviation above the population mean. Given that 0.5 s.d. represents a small absolute departure from baseline, the discrepancy between our results and those of Kiire may not be meaningful. Alternatively, it could reflect cross-cultural variation: we sampled heterosexual females from the public in the US whereas Kiire [34] used a mixture of male and female Japanese undergraduates; there may be geographical variation in limb-length preferences reflecting local environmental factors, but this has not yet been investigated with high-quality, detailed figures [35,37,38]. A second study used three-dimensional-figurines to investigate the developmental trajectory of LBR preference among Polish children and teenagers, using a small set of widely-spaced stimuli (−15%, −7.5%, 0%, +7.5%, and +15% above baseline) [36]. The authors report little preference among younger participants, but state that for those aged 15–20 the most attractive LBR was 7.5% above the approximate population average—a result which is directionally the same as ours but more extreme, although the small number of tested LBRs and rather limited information about the data analysis and relation between the tested LBRs and population distribution makes direct comparison with our work difficult. One additional study studied female LBRs using three-dimensional-rendered images and reported a quadratic trend, such that extreme values were less attractive, but did not attempt to identify the peak of the judgement function [40].

Our finding of a preference for slightly-above-average LBRs may reflect a trade-off. On the one hand, very long limbs may indicate harmful genetic conditions [30] and, more generally, averageness is thought to signal genetic diversity and immunocompetence [21]. For example, human males with more average-looking faces have greater heterozygosity in the major histocompatibility complex [54], corresponding to immunity against a wider range of pathogens [55]. However, relatively long legs are thought to indicate ‘reserve capacity’ that can buffer against nutritional or mechanical stress [22]; moreover, since environmental stress affects leg growth more than body size, higher LBR indicates stable development and general welfare [56]. The optimally-attractive LBR may therefore reflect a compromise between these factors. This suggestion is tentative, however, because our image-format results indicate the sensitivity of LBR-attractiveness ratings to superficial stimulus features. Although we used rendered images based on anthropometric data, the stimuli are still less naturalistic than those obtained from three-dimensional body scans, and are notably more muscular than typical US males. Likewise, although we found little difference between the three non-silhouette formats—in particular, the results were similar for the figures with White (Caucasian) and Black (e.g. African-American) colouring—it will be important to test this with photo-realistic skin-tones. In addition, we focused on heterosexual females, and our studies were not designed to investigate whether the effects of LBR or image format are modulated by participant characteristics such as age or ethnic group.

All of these considerations provide important directions for future work, and we suggest that it would be useful for such work to incorporate the methodological innovations applied here. Nonetheless, our studies indicate that the effects of limb length on attractiveness judgements vary with stimulus format, and that, for realistic computer-generated images of male figures, the optimally-attractive leg-to-body ratio is slightly greater than the population average.

## Supplementary Material

The effect of leg-to-body ratio on male attractiveness depends on the ecological validity of the figures

## Data accessibility

The data reported in this paper are included as the electronic supplementary material.
